# The Effect of Topical 0.05% Cyclosporine in the Prevention of Recurrence Following Pterygium Surgery

**DOI:** 10.14744/bej.2021.68442

**Published:** 2021-09-27

**Authors:** Ayse Sevgi Karadag, Emre Guler, Emre Guler

**Affiliations:** 1. Department of Ophthalmology, Adıyaman University Training and Research Hospital, Adıyaman, Turkey; 2. Department of Ophthalmology, Turkiye Hospital, Istanbul, Turkey; 3. Prof. Dr. N. Resat Belger Beyoglu Eye Training and Research Hospital, Istanbul, Turkey

**Keywords:** Limbal-conjunctival autograft, pterygium, pterygium recurrence, Schirmer I, topical cyclosporine A

## Abstract

**Objectives:**

To evaluate the effect of topical cyclosporine A (CsA) (Restasis, Abbvie, Lake Bluff, IL, USA) on recurrence rates and tear metrics after pterygium excision and limbal-conjunctival autografting (LCUA) technique in patients with primary pterygium.

**Methods:**

A total of 60 eyes of 60 patients with primary pterygium who underwent pterygium excision and the LCUA technique were evaluated prospectively. Among them, 30 eyes of 30 patients were treated with topical CsA (Restasis) for 6 months postoperatively (Group 1). The remainder of the patients were assigned to Group 2. The follow-up period was 12 months for each group. The primary outcome measures were a comparison of Schirmer I test and fluorescein tear break-up time (FTBUT) results, the recurrence rate, and postoperative complications.

**Results:**

There were no significant differences in age (mean age of Group 1 and 2 was 55.0±9.7 years and 56.3±8.9 years, respectively) or sex between groups (p>0.05). Farmers were the largest group of patients (40.0%). Recurrence of pterygium was observed in 5 (16.6%) eyes in Group 1 and 8 (26.6%) eyes in Group 2. The recurrence rate was not statistically significant between groups (p=0.35). The complication rate was significantly lower in Group 1 compared with that of Group 2 (p=0.02). The average increase in Schirmer I and FTBUT values was significantly higher in Group 1 than in Group 2 (p<0.05).

**Conclusion:**

The use of topical CsA did not demonstrate any significant improvement in the recurrence rate of pterygium following LCUA surgery.

## Introduction

Pterygium is one of the most common ocular surface diseases associated with neovascularization and inflammation (1). The major accepted etiology for the disease is the chronic ultraviolet radiation exposure which leads to the secretion of pro-inflammatory cytokines (2). Classically wing-shaped lesion stretches to the center of the cornea and causes irritation and visual loss related to the occlusion of visual axis and induced astigmatism (3). The treatment of the disease is still surgical excision of the lesion; however, it has the major drawback of postoperative recurrence (4). Various adjuvant therapies (5, 6) or surgical modifications (7) were proposed for the prevention of pterygium recurrence.

Cyclosporine A (CsA) is an immunosuppressive agent that selectively inhibits T-helper cells and prohibits the expression of pro-inflammatory cytokines (8). In addition, it has been shown to inhibit angiogenesis against the vascular endothelial growth factor (VEGF) (8, 9). Topical CsA has been approved for the treatment of dry eye syndrome (DES), however, it is used for a variety of ocular conditions such as vernal keratoconjunctivitis (10), corneal transplants (11), corneal ulcers (12), and herpetic stromal keratitis (13). Several recent reports have shown that clinical use of CsA inhibits pterygium recurrence after surgery due to its inhibitory effect on migration of pterygium fibroblast and expression of matrix metalloproteinases (14-18).

Various methods are available for the investigation of DES. Among these Schirmer test or fluorescein break up-take time (FTBUT) have been used in several studies to investigate the relationship between pterygium and DES with conflicting results. In this study, we investigated the effectiveness of topical CsA on the patients who underwent pterygium excision with limbal-conjunctival autografting (LCAU) and then compare the incidence of recurrence in pterygium after the surgery for the treatment and control groups.

## Methods

In this prospective study, 60 eyes of 60 patients with primary pterygium who underwent pterygium excision using the LCAU technique were included in the study. The patients were randomly divided into two groups (Group 1 and Group 2), both including 30 eyes of 30 patients. All the included patients had primary nasal pterygium exceeding 2 mm (the lesion was measured horizontally from limbus to cornea with slit-lamp examination), ocular irritation resistant to medical treatment, and decreased visual acuity associated to the pterygium. Exclusion criteria were recurrent and atrophic pterygium, topical CsA allergy, history of the lid or ocular diseases, use of systemic or local medication, and pregnancy.

One experienced surgeon performed all surgeries (ASK). Under local anesthesia with 0.5% proparacaine hydrochloride, the pterygium head was removed from the cornea with a scalpel; then, together with Tenon’s capsule, the pterygium was dissected with Westcott scissor. In cases of bleeding, cauterization was avoided in order to prevent any damage to the limbal area. Under the supratemporal bulbar conjunctiva, 0.25 ml of normal saline was injected, and a piece of the conjunctiva, equal in size to the bare area left at the site of the excised pterygium, was marked from the limbal region and removed from Tenon’s capsule. We performed a superficial circumferential incision 0.5-mm from the superotemporal limbus to include limbal epithelium in the conjunctival graft. This piece of the conjunctiva was then sutured, with a continuous 10/0 nylon suture, to the bare area. The region where the conjunctival autograft was taken from was left for primary healing. All the patients received topical antibiotic (moxifloxacine) four times daily for 2 weeks, and topical steroids (dexamethasone) four times daily for a month. Topical 0.05% CsA (Restasis^®^, Allergan Pharmaceutical) was given to Group 1, with 6-h periods in a day during the postoperative 6 months. In Group 2, patients did not receive CsA.

Pre- and post-operatively, each patient underwent a standard ophthalmological examination that included uncorrected visual acuity, BCVA, intraocular pressure ,and slit-lamp biomicroscopy. In addition, measurement of FTBUT and the Schirmer I test were performed by the same investigator for each patient. The study was conducted in accordance with the ethical standards stated in the Declaration of Helsinki. The study was approved by the Local Ethics Committee of the participating center. All patients were informed about the purpose of the study and provided their consent.

Main outcome measures were the comparison of FTBUT and Schirmer I test results, recurrence rates, and postoperative complications between groups. The lesion was defined as recurrent when the fibrovascular tissue covered the corneal surface higher than 1 mm (3, 19).

Data were encoded and analyzed using the Statistical Package for Social Sciences (SPSS) software (version 21.0, SPSS, Inc.). The data were not normally distributed, met by the Kolmogorov–Smirnov test (p<0.05). Quantitative data were compared by Mann–Whitney U and Wilcoxon Signed Ranks Test tests. Kaplan–Meier survival analysis was performed to determine the recurrence-free survival time. Chi-square test was used to investigate the categorical data. P-value <0.05 were considered to be statistically significant.

## Results

The mean age of Group 1 and 2 was 55.0±9.7 and 56.3±8.9 years, respectively (p=0.54). A total of 14 (46.6%) of the patients in the treatment group were male; 16 (53.3%) of the patients in the control group were male (p=0.45). The mean follow-up period for Group 1 was 11.6±3.4 months (range, 8–16) and 12.7±3.2 months (range, 8–15) for Group 2 (p=0.21). A total of 24 (40.0%) of the patients included in our study were farmers, 13 of whom were Group 1, and the reminder in Group 2. A total of 23 (38.3%) were housewives, 13 (21.6%) were civil servants.

In Group 1, the mean preoperative Schirmer I and FTBUT values were 7.9±2.6 mm and 7.7±2.0 s. At the end of the follow-up period, these were 12.3±1.9 mm and 13.7±1.9 s, respectively. In Group 2, the mean preoperative Schirmer I and FTBUT values of Group 1 were 10.6±2.5 mm and 10.0±3.3 s. At the last visit, these were 13.5±2.8 mm and 12.9±3.0 s, respectively. The mean postoperative Schirmer I and FTBUT were not significantly different between groups (p>0.05). However, when pre-and post-operative Schirmer I and FTBUT values were compared, the average increase was significantly higher in Group 1 (p<0.05) (Table 1).

**Table 1.** FBUT and Schirmer I test results within the groups during the follow-up period

	**tCsA applied**	**tCsA not applied**	**p-value**
	**(mean±SD)**	**(mean±SD)**	
FTBUT (second)			
Preoperative	7.7±2.0	10.6±2.5	<0.01
Postoperative 12 months	13.7±1.9	13.5±2.8	0.80
Change	5.9±1.8	2.9±2.7	<0.01
Schirmer I (mm)			
Preoperative	7.9±2.6	10.0±3.3	<0.01
Postoperative 12 months	12.3±1.9	12.9±3.0	0.86
Change	4.4±1.8	2.9±3.0	0.02

FTBUT: Fluorescein Tear Break up-take time; SD: Standard deviation; tCsA: Topical cyclosporine A. Mann-Whitney U test was applied. P<0.05 indicates statistically significance.

In the early postoperative period, all patients in the two groups had symptomatic complaints of ocular pain, photophobia, lacrimation, and sensation of a foreign body in the eye. No adverse effect was observed in patients receiving topical CsA except a mild irritation when the drug was applied. Punctate corneal staining was not detected in Group 1. No serious complication has been detected in any of the cases in our study.

The only postoperative complication in our study was Dellen formation. This complication was significantly higher in Group 2 (8 patients, 26.6%) compared to Group 1 (2 patients, 6.6%) (p=0.02). The Dellen formation was healed after treatment with preservative-free eye drops within 1 week.

The mean recurrence time was 2.40±1.16 months in Group 1 and 3.00±0.70 months in Group 2. Recurrence of pterygium was observed in 5 (16.6%) eyes in Group 1 and 8 (26.6%) eyes in Group 2. The risk of pterygium recurrence was 1.6 times higher in Group 2 than in Group 1 (OR=1.600, 95% CI=0.591–4.333). However, the difference was not statistically significant between groups (p=0.57).

## Discussion

The treatment for pterygium is surgical excision. Although bare sclera excision is a simple approach, recurrence is the most common complication. However, there is no definitive treatment modality that prevents postoperative recurrence as well as associated complications.

A previous research demonstrated that pterygium is a local deficit of limbus (20). The degradation of the limbal barrier leads to the proliferation of the pterygium onto the cornea (21). Al Fayez (19) showed that the recurrence rates were not statistically different between conjunctival and LCAU transplantation, however, LCAU was more efficient in recurrent pterygium. In the current study, we used the LCAU technique, which has been proved to be safe and effective. In our cases, we used continuous sutures for the stabilization of the graft. However, interrupted sutures may provide better graft tension and may result lower recurrence rates but these should be evaluated in future studies.

Adjuvant agents are treatment choices that help to eliminate recurrence after surgery. Mitomycin C (MMC) is the most commonly used adjuvant agent that prevents cellular activity by inhibiting DNA synthesis (6). It has anti-proliferative effect and prevents recurrence of the pterygium. Previous studies showed recurrence rates varying from 3% to 38% in primary pterygium when MMC was used intraoperatively (22-25). However, the use of MMC may lead to severe ocular complications including scleral thinning and necrosis, corneal decompensation, and glaucoma (26, 27). In our study, the recurrence rates were 16.6% in Group 1 and 26.6% in Group 2. Group 1 achieved similar recurrence rate to MMC avoiding its ocular side effects.

The CsA is anti-inflammatory drug that has been widely applied topically in ocular surface diseases (10-13). Previously, it has been demonstrated to inhibit the expression of pro-inflammatory cytokines including tumor necrosis factor-α and also prevents angiogenesis associated to VEGF (8, 9). Another study demonstrated that CsA inhibits the migration of fibroblasts in pterygium tissue in terms of blocking the expressions of matrix metalloproteinase-3 and -13 (17). The CsA was fairly effective in the inhibition of fibroblast migration despite the short application period and low dosage. These findings reveal that postoperative topical CsA administration can be effective to avoid recurrences following primary pterygium excision.

Several studies evaluated the use of topical CsA to prevent recurrences following pterygium excision (Table 2) (18, 28-32). In all these studies topical CsA was found to be effective in preventing pterygium recurrence. Among these the effect of topical 0.05% CsA has been evaluated in patients that underwent the same surgical technique by Aydin et al. (30) They found that the application of topical CsA following LCAU surgery was effective in preventing recurrence, ocular discomfort, and complications. Their recurrence rate was 3.4%. In the current study, the recurrence rate was 26.6% for LCAU and 16.6% for LCAU technique + topical 0.05% CsA. However, different from the previous studies, the recurrence rates were not statistically significant between groups. In addition, no serious complications occurred in any of the patients as well as decreased postoperative recurrence rate.

**Table 2.** Study outcomes reporting topical CsA use after pterygium surgery

Study	Total eyes	Follow-up time	Primary outcomes	Results
(year)	(n)	(months)
Özülken et al. (26)	26	11	Recurrence rate,	The recurrence rates were significantly lower in CsA
(2012)			Complications	group (23%) than control group (46%)
				Complications were significantly lower in the CsA group.
Vural et al. (27)	18	12	Recurrence rate	The recurrence rates were significantly lower in CsA
(2011)				group (22%) than control group (44%)
qIbáñez et al. (18)	40	6	Recurrence rate,	The recurrence rates were significantly lower in CsA
(2009)			Complications	group (7.5%) than control group (17.5%)
				No patients had postoperative complications in the CsA group. These occurred in 10 eyes in the control group.
Aydin et al. (28)	30	12	Recurrence rate,	The recurrence rates were significantly lower in CsA
(2008)			Postoperative pain using the visual analog scale (VAS), Complications	group (3.4%) than control group (17.9%)
				The average VAS was significantly lower in the CsA group (p=0.034)
				The ratio of the cases with no complications was statistically significantly higher in the CsA group (p=0.017).
Tok et al. (29)	31	12	Recurrence rate	The recurrence rates were significantly lower in CsA
(2008)				group (12.9%) than control group (45.2%)
Wu et al.(30)	25	10	Recurrence rate	The recurrence rates were significantly lower in CsA
(1999)				group (5%) than control group (10%)

Although the association between DES and pterygium is unclear, some studies suggested that the alteration of tear film layer may induce the proliferation subconjunctival fibrovascular tissue. Gunduz et al. (13) reported lower Schirmer I and FTBUT results in patients with pterygium than controls and suggested that DES may be associated with the development of pterygium.

In our study, we aimed to evaluate whether the use of topical CsA following pterygium surgery has any effect on Schirmer I and FTBUT test results which may be the indicators of DES. Postoperatively, these values were not significantly different between groups however, the average increase in these values was significantly higher in Group 1. Although the patients treated with topical CsA did not show any significant recurrence rates, topical CsA may help to correct tear film layer irregularities and consequently treats DES following pterygium surgery which contributes to decrement of recurrence rates in addition to its anti-inflammatory effect.

The CsA 0.5% formulation twice a day dosing was approved for clinical use based on the phase 3 studies. The ocular retention time of this formulation was approximately 2 h hence a more frequent dosing might be necessary in cases with severe dry eye or ocular surface disorders to control the inflammation more effectively (33, 34). In addition, the repeated use of this formulation leads to an increased accumulation of CsA in the conjunctiva, cornea, and lacrimal gland (35). Application of topical CsA 0.05% at higher than approved dosages (up to 8 times a day) has shown acceptable tolerability and local good efficacy in patients with severe ocular surface diseases (36). Hence, we used a four times a day regimen for CsA rather than the regular, efficient dose as twice a day.

Dellen was the main postoperative complication in the current study. This may be explained by corneal irregularity due to decreased Schirmer I results in the early postoperative period. These patients were successfully treated with preservative-free eye drops.

This study has some limitations. First is the small sample size. Second is the short follow-up period. Third is the lack of pterygium size information since higher level of corneal involvement is correlated with higher recurrences. Finally, there was no placebo treatment for Group 2.

In conclusion, topical CsA did not show any significant improvement in the recurrence rate of pterygium following LCAU surgery. Our findings should be evaluated in future prospective studies including a larger population in a long follow-up period.

## Disclosures

### Ethics Committee Approval:

Adiyaman University Faculty of Medicine Biomedical Research Ethics Committee, protocol number: 2015/1-9, Date: 25/01/2015.

### Peer-review:

Externally peer-reviewed.

### Conflict of Interest:

None declared.

### Authorship Contributions:

Involved in design and conduct of the study (ASK, EG, SG); preparation and review of the study (ASK, EG, SG); data collection (ASK, EG, SG); and statistical analysis (ASK, EG, SG).
